# Analysis of the Necrosis-Inducing Components of the Venom of *Naja atra* and Assessment of the Neutralization Ability of Freeze-Dried Antivenom

**DOI:** 10.3390/toxins13090619

**Published:** 2021-09-02

**Authors:** Cheng-Hsuan Ho, Liao-Chun Chiang, Yan-Chiao Mao, Kuo-Cheng Lan, Shih-Hung Tsai, Yu-Jen Shih, Yuan-Sheng Tzeng, Chin-Sheng Lin, Wen-Loung Lin, Wei-Hsuan Fang, Kuang-Ting Chen, Chi-Hsin Lee, Dapi Meng-Lin Chiang, Shing-Hwa Liu

**Affiliations:** 1 Department of Emergency Medicine, Tri-Service General Hospital, National Defense Medical Center, Taipei 114, Taiwan; erdoctorho@gmail.com (C.-H.H.); kclan.tw@yahoo.com.tw (K.-C.L.); tsaishihung@yahoo.com.tw (S.-H.T.); 2 Institute of Toxicology, College of Medicine, National Taiwan University, Taipei 100, Taiwan; 3 College of Life Sciences, National Tsing Hua University, Hsinchu 300, Taiwan; axe956956@gmail.com; 4 Institute of Biology and Anatomy, National Defense Medical Center, Taipei 114, Taiwan; 5 Division of Clinical Toxicology, Department of Emergency Medicine, Taichung Veterans General Hospital, Taichung 407, Taiwan; doc1385e@gmail.com; 6 Division of Plastic Surgery, Department of Surgery, Tri-Service General Hospital, National Defense Medical Center, Taipei 114, Taiwan; yorky@mail.ndmctsgh.edu.tw (Y.-J.S.); m6246kimo@yahoo.com.tw (Y.-S.T.); 7 Division of Cardiology, Department of Internal Medicine, Tri-Service General Hospital, National Defense Medical Center, Taipei 114, Taiwan; littlelincs@gmail.com; 8 Taichung Wildlife Conservation Group, Taichung 436, Taiwan; ketupaflavpes@yahoo.com.tw (W.-L.L.); serpentes2019@gmail.com (W.-H.F.); 9 Department of Chinese Medicine, Chang Bing Show Chwan Memorial Hospital, Changhua 505, Taiwan; U102022410@cmu.edu.tw; 10 School of Medical Laboratory Science and Biotechnology, College of Medical Science and Technology, Taipei Medical University, Taipei 110, Taiwan; chihsine@msn.com; 11 Division of Animal Physiology and Immunology, TUM School of Life Sciences Weihenstephan, Technical University Munich, 85354 Freising, Germany; dapi_chiang@biovesicle.com; 12 Department of Medical Research, China Medical University Hospital, China Medical University, Taichung 404, Taiwan; 13 Department of Pediatrics, College of Medicine, National Taiwan University Hospital, Taipei 100, Taiwan

**Keywords:** *Naja atra*, snake, minimum necrotizing dose

## Abstract

Patients bitten by *Naja atra* who are treated with bivalent freeze-dried neurotoxic antivenom in Taiwan have an improved survival rate but develop necrotic wound changes. The World Health Organization (WHO) has suggested using the minimum necrotizing dose (MND) of venom as a method of evaluating the neutralization effect of antivenom. The aim of this study was to evaluate the effectiveness of antivenom for the prevention of necrosis based on the MND and clarify which component of the venom of *N. atra* induces necrosis. The neurotoxins (NTXs) were removed from the crude venom (deNTXs), and different concentrations of deNTXs were injected intradermally into the dorsal skin of mice. After three days, the necrotic lesion diameter was found to be approximately 5 mm, and the MND was calculated. A reduction in the necrotic diameter of 50% was used to identify the MND_50_. Furthermore, both phospholipase A_2_ (PLA_2_) and cytotoxins (CTXs) were separately removed from the deNTXs to identify the major necrosis-inducing factor, and the necrotic lesions were scored. All mice injected with deNTXs survived for three days and developed necrotic wounds. The MND of the deNTXs for mice was 0.494 ± 0.029 µg/g, that of the deNTXs-dePLA_2_ (major component retained: CTXs) was 0.294 ± 0.05 µg/g, and that of the deNTX-deCTX (major component retained: PLA_2_) venom was greater than 1.25 µg/g. These values show that CTX is the major factor inducing necrosis. These results suggest that the use of the deNTXs is necessary to enable the mice to survive long enough to develop venom-induced cytolytic effects. CTXs play a major role in *N. atra*-related necrosis. However, the MND_50_ could not be identified in this study, which meant that the antivenom did not neutralize venom-induced necrosis.

## 1. Introduction

*Naja atra*, a member of the Elapidae family, is a medically significant venomous snake that is common in Central Taiwan [1]. Patients bitten by *N. atra* are treated with bivalent antivenom-freeze-dried neurotoxic antivenom in Taiwan [2]. Local injuries are more common than neurologic toxicity after bites from many *Naja* species, including *Naja nigricollis, Naja mossambica, Naja nigricincta, Naja pallida, Naja nubiae, and Naja katiensis* [3,4,5]. Among patients bitten by *N. atra*, 65.6% progress to skin necrosis, and 42.1% develop necrotizing soft tissue infections [1,6]. Even when patients receive antivenom, skin necrosis still occurs, and debridement surgery is usually suggested 3.5 days after snake bite [6]. Studies have investigated the mechanism for *N. nigricollis* [7,8] and *N. atra* [9], although there has been less research on the latter species.

Snakebite-related local toxicity is usually thought to be due to the action of phospholipase A_2_ (PLA_2_), cytotoxins (CTXs, also called cardiotoxins) and snake venom metalloproteinases (SVMPs) [10,11,12]. PLA_2_ disrupts the plasma membrane of muscle fibers and induces a signaling cascade, including a calcium influx and mitochondrial dysfunction, resulting in muscle cell damage [13,14]. CTXs are unique to the Elapidae family and induce extensive local injuries by disrupting the plasma membranes of different cells [5,15]. SVMPs are the major component of the venom of most Crotalinae and Viperinae species [16] and are less abundant in the venom of Elapidae species [10,17]. The venom of *N. atra* comprises neurotoxins (NTXs), PLA_2_, CTXs, and high-molecular-weight proteins (HMWPs) [18] which included Atrase B [19], Atragin [20], kaouthiagin-like [20], and L-amino acid oxidase (LAAO) [21]. In Taiwan, the composition of the venom of *N. atra* varies across its geographic range, particularly between Western and Eastern Taiwan [18]. The average component ratio of the crude venom of *N. atra* is as follows: NTXs, 22%; PLA_2_, 15.4%; CTXs, 56.2%; and other components, 6.5% [11,18]. Therefore, PLA_2_ and CTXs are suspected of being the major causes of the local injuries induced by *N. atra* bites.

A significantly improved survival rate has been observed among patients bitten by *N. atra* who receive antivenom; however, these patients still have a high likelihood of developing local necrotic wounds [6]. Dermonecrosis in humans caused by the venom of *N. atra* is an important clinical problem even in the era of antivenom. Traditionally, antivenom is evaluated based on the effective dose 50 (ED_50_), which is based on the lethal dose 50 (LD_50_) of crude venom in mice [22,23]. In summary, the effectiveness of traditional antivenom is evaluated based on the improvement in the survival rate and does not take into account cytolytic effects. Therefore, the World Health Organization (WHO) suggested using the minimum necrotizing dose (MND) of venom as a method of evaluating the neutralizing effect of antivenom [24]. The MND of venom is the smallest dose that, when injected intradermally into the dorsal skin of mice, leads to the development of a necrotic lesion 5 mm in diameter [24]. The MND_50_ is the value used to evaluate the neutralizing effect of antivenom on venom-induced necrosis [24]. There were other studies focused on the *Naja* genus-related local injury [5,7,9]. Our study separated each venom component performed as previously described [18] and approached with MND, MND50, and tissue necrosis score to identify the effects of venom and antivenom.

The aim of this study was to evaluate the effectiveness of antivenom with regard to the prevention of necrosis based on the MND/MND_50_ and to identify which component of the venom of *N. atra* leads to necrosis.

## 2. Results

### 2.1. An Example for the Clinical Observation of Patient Bitten by N. atra

**Figure 1:**
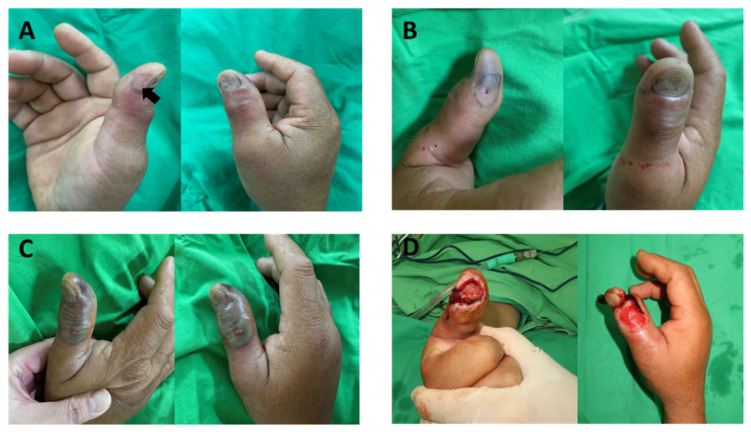
A 51-year-old male was bitten over the right thumb distal phalanx by *N. atra*, which was identified by the patient. (**A**) Nine hours post-bite, the fang maker (arrow) was located over the radial side, and redness was located over the dorsal side of the thumb. (**B**) Fifteen hours post-bite, the necrotic change can be noted over the fang marker, and the redness is still noted over the dorsal side of the thumb. (**C**) Fifty-seven hours post-bite, the progress of necrosis was noted from the fang marker and progressing to dorsal side. (**D**) (Photo credits: *Yu-Jen Shih*) Ten days post-bite, the patient received the third time of debridement, and the necrotic tissue was all debrided and removed.

A 51-year-old male who was bitten by *N. atra*, which was identified by the patient, received one vial of the bivalent antivenom against *B. multicinctus* and *N. atra* and developed progressive necrosis without any neurologic symptoms two days later (Figure 1). He underwent debridement several times and remained in the hospital for twenty-two days. The wound cultures showed infection with *Morganella morganii* and Enterococcus faecalis, which were commonly identified in patients who have been bitten by *N. atra* [25]. Patients bitten by *N. atra* developed delayed necrotic wounds, and an appropriate animal mode was necessary for mice to survive long enough to develop the cytolytic effects.

### 2.2. Characterization of Naja atra Crude Venom and the MND of the deNTXs

**Figure 2:**
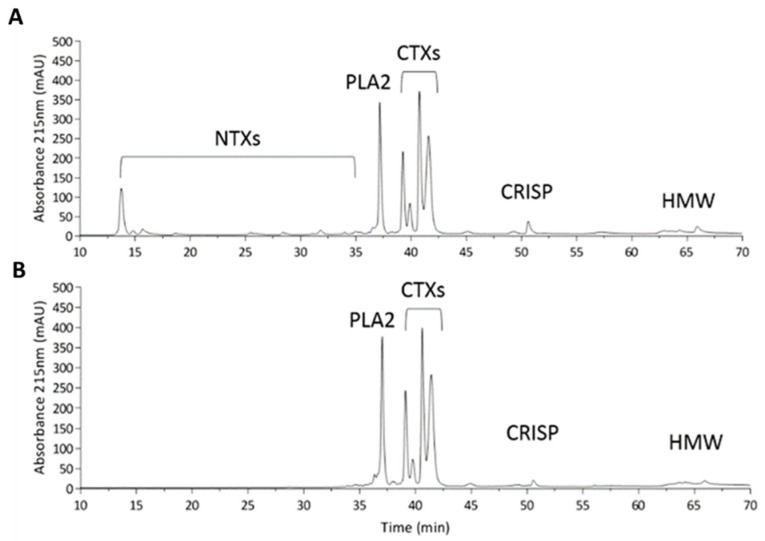
Characterization of *Naja atra* crude venom and crude venom devoid of NTXs. (**A**) HPLC profile of the *N. atra* crude venom sample and (**B**) *N. atra* crude venom devoid of NTXs (deNTXs). One hundred micrograms of both samples were applied to a Phenomenex Jupiter^®^ C18 column (250 × 4.6 mm, 5 µm particle size, 300 Å pore size) for analysis. Abbreviation: NTXs, neurotoxins; PLA_2_, phospholipase A_2_; CTXs, cytotoxins also called cardiotoxins; CRISP, cysteine-rich secretory protein; HMW, high-molecular-weight proteins.

**Figure 3:**
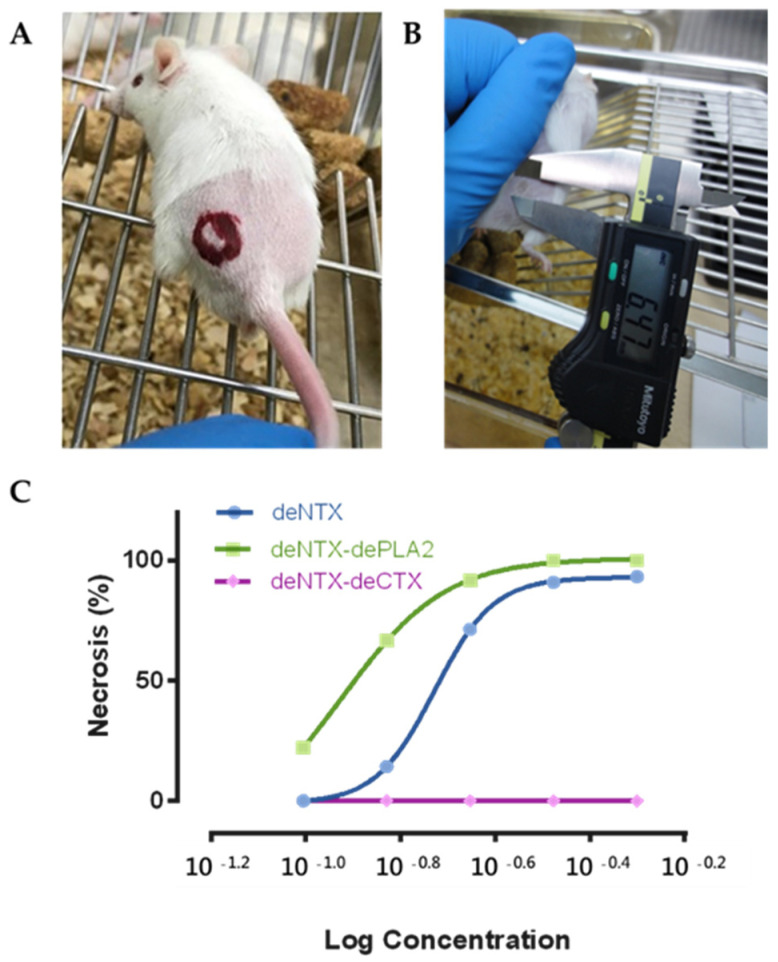
Male CD-1 mice (10–12 weeks old, 20–22 g) had (**A**) the dorsal skin hair removed and were intradermally injected with different venoms, and then, (**B**) the necrosis diameter was measured after three days. (**C**) The MNDs of different venoms (deNTXs, blue curve; deNTXs-dePLA_2_, green curve; deNTXs-deCTX, purple curve) were determined with linear interpolation.

The crude venom of *N. atra* was found to contain NTXs, PLA_2_, CTXs, cysteine-rich secretory proteins (CRISPs) and high-molecular-weight proteins (HMWPs) (Figure 2A). We removed the NTXs (the lethal component) from the crude venom, creating venom devoid of NTXs (deNTXs) (Figure 2B). All mice that were intradermally injected (Figure 3) with the deNTXs survived for three days, and necrotic changes were observed, as in human wounds. Different concentrations of deNTXs were tested, and the MND of the deNTXs in mice was 0.494 ± 0.029 µg/g (injection volume: 50 µL, mouse weight: 20~22 g).

### 2.3. CTXs Are the Major Component Causing Necrosis

Furthermore, we removed the CTXs and PLA_2_ separately from the deNTXs to determine which component played the most important role in inducing necrosis (Figure 3C). The MND of deNTXs-dePLA_2_ (major component retained: CTXs) was 0.294 ± 0.050 µg/g, and the MND of deNTXs-deCTXs (major component retained: PLA_2_) was greater than 1.25 µg/g (Table 1). The results showed that CTXs played a major role in the mechanism generating necrosis.

### 2.4. Development of Necrosis

**Figure 4:**
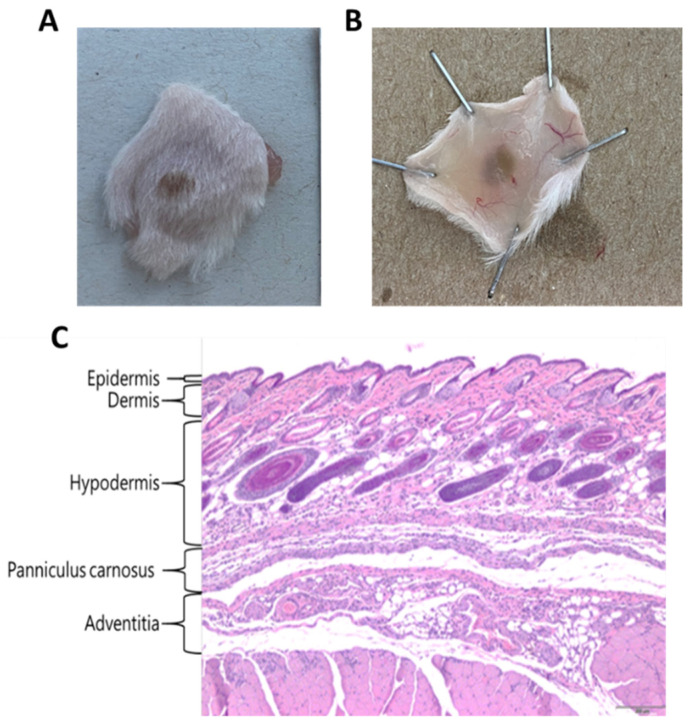
The mice were injected intradermally with different venom levels of 0.5, 0.33, 0.22, 0.148, and 0.098 µg/g after 72 h. We removed the dorsal skin, and biopsy was performed with HE staining. (**A**): Dorsal necrotic lesion (**B**): The inside of the necrotic lesion (**C**): The mice were intradermally injected with deNTXs (0.22 µg/g). A diffuse distribution and loose organizational integrity (necrosis score of 4) over the panniculus carnosus and adventitia were observed.

**Figure 5:**
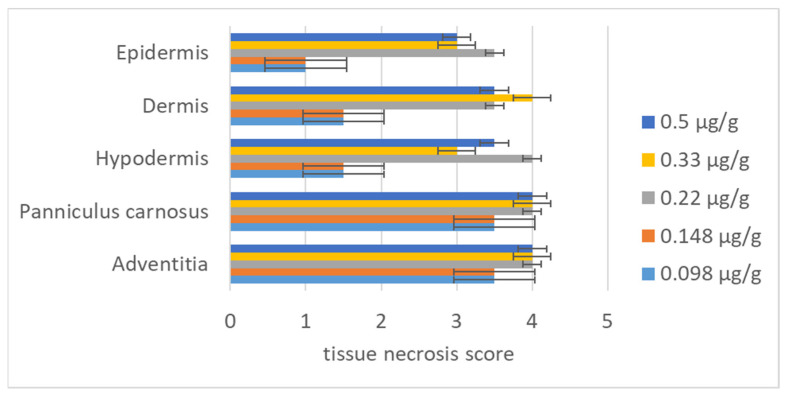
A veterinary pathologist scored the necrotic lesion layer-by-layer in the dorsal skin intradermally injected with different deNTXs doses (0.5; 0.33; 0.22; 0.148, and 0.098 µg/g) (each group 6 mice) three days later.

After determining the MND, we established a series of low concentrations of deNTXs to investigate the mechanism underlying the development of necrosis. Mice were injected intradermally with different concentrations (0.5, 0.33, 0.22, 0.148, and 0.098 µg/g), and the necrotic changes were observed after 72 h. We removed the necrotic skin and sent it for biopsy. The necrosis was scored by a veterinary pathologist. The severity of the necrosis was classified as normal (score of 0), minimal (score of 1), mild (score of 2), moderate (score of 3), or severe (score of 4) (Table 2). In the biopsy, severe necrosis appears as the loss of organization and substantial increase in tissue space. The skin biopsies were evaluated in the individual layers, namely the epidermis, dermis, hypodermis, panniculus carnosus and adventitia (Figure 4). Even when the dose was less than the MND (0.494 ± 0.029 µg/g), the deNTXs at 0.098 µg/g still induced necrosis, and the pathology extended deeper than the dermis. The most severely destroyed layer was the panniculus carnosus and adventitia, even at the minimum dose (Figure 5 and Supplementary Table S1).

### 2.5. Neutralization Ability of the Antivenom

**Figure 6:**
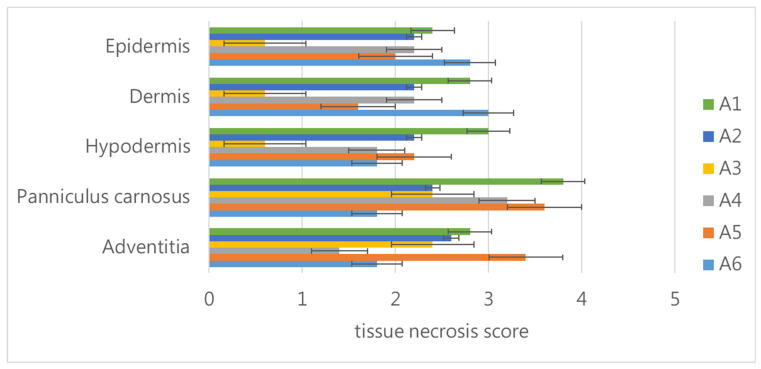
The mice (each group 5 mice) were injected intradermally with a mixture of the challenge dose (two times the MND of deNTXs) and different dilutions of antivenom (A1, original; A2 1:1; A3 1:2; A4 1:3; A5 1:4 and A6 1:5). After three days, the veterinary pathologist scored the necrotic lesion layer-by-layer in the dorsal skin.

We used two times the MND of the deNTXs as the challenge dose. We mixed this with different dilutions of antivenoms in vitro and then injected the mixtures intradermally into each mouse (5 mice per group). We compared the results with those in mice injected with a challenge dose mixed with saline to identify the MND_50_, as per the recommendation of the WHO [24]. The mice injected with the challenge dose mixed with saline developed necrotic lesions with diameters of approximately 7 mm. The other mice injected with different dilutions of antivenom (from the original to 1:5 dilution) still had necrotic lesion diameters that were greater than 5 mm. None of the mice developed necrotic lesion diameters that were 50% smaller than those in the mice injected with two times the MND mixed with saline. Therefore, we could not identify the MND_50_ with this antivenom. The necrotic lesions were biopsied, and the necrosis was scored in the individual skin layers (Figure 6). Regardless of the concentration of antivenom, including the original concentration, the challenge dose of the deNTXs resulted in necrosis throughout all of the layers of the skin, including severe necrosis in the panniculus carnosus and adventitia.

## 3. Discussion

The traditional method of evaluating the antivenom effect with the LD_50_ and ED_50_ may not be suitable for the assessment of venom-related cytolytic effects. Mice injected with the crude venom of *N. atra* would not survive long enough to develop the necrotic wounds seen in humans [9]. NTXs are the most lethal component of *N. atra* venom [12]. NTXs have a strong affinity for the mouse post-synaptic acetylcholine receptor (AchR) and block neuromuscular function, resulting in acute toxicity, including tremors, rigidity and even death; this is the main mechanism leading to death [11,26]. We suspected that the NTXs in the venom of *N. atra* might have relatively low affinity for human neuroreceptors, or that there was another mechanism that remained unclear that explained why people bitten by *N. atra* develop few neurologic symptoms [6]. In people who have been bitten by *N. atra*, the NTXs do not lead to immediate mortality, which gives the other toxins enough time to cause necrosis. Patients bitten by *N. atra* developed delayed necrotic wounds, and the deNTXs were necessary for mice to survive long enough to develop the cytolytic effects.

The LD_50_ of the crude venom injected intravenously into adult mice was 0.56 µg/g [27]. The NTXs are the lethal component of the crude venom, and the LD_50_ of the NTXs intravenously injected was 0.075 µg/g [12]. After the NTXs were removed, the MND of the deNTXs was found to be 0.494 ± 0.029 µg/g. Evaluating the neutralizing ability of antivenom on the LD_50_ in mice alone is not adequate with regard to necrosis. The MND identifies the necrotic effect of the venom, and the MND_50_ refers to the anti-necrosis effect of the antivenom [24].

Both CTXs and PLA_2_ are suspected of being the major components of the venom of *N. nigricollis* causing dermonecrosis [5]. In this study, we identified that CTXs played a major role in the mechanism by which the venom of *N. atra* caused necrosis. CTXs are less toxic than NTXs, although they have cytotoxic effects that contribute to the lethality of the venom [12]. There are many subtypes of CTXs, including types I, II, III, and IV, and all have cytolytic activity [12]. CTXs can penetrate cell membranes by damaging the phospholipid bilayer [28]. CTXs have a positive charge and easily bind to membranes and vesicles, which have negative charges, and tighter binding leads to increased cellular lysis [26]. PLA_2_ can be classified as belonging to group I (Elapidae) and group II (Viperidae) [29]. In addition, PLA_2_ is also thought to play a role in local muscle injury. In this study, significant variation was noted in comparing the MND of deNTXs-dePLA_2_ (major component retained: CTXs) with the MND of deNTXs-deCTXs (major component retained: PLA_2_) (0.294 ± 0.050 µg/g vs. greater than 1.25 µg/g). We suspected that necrosis was mainly caused by CTXs, and there were no significant synergistic effects of CTXs and PLA_2_. Similarly, the use of a PLA_2_ inhibitor did not decrease the necrotic area caused by the venom of *N. nigricollis* [5]. However, whether there was a synergistic effect of NTXs could not be investigated in this study.

The fangs of *N. atra* are noticeably short [1,30]. We used intradermal injections to simulate the real-world condition. As observed in the biopsy specimen, the venom was injected into the intradermal layer, after which the venom penetrated deeper rather than spreading along the surface. The deNTXs was distributed throughout the soft tissue rather than remaining localized at the site of injection, and severe damage was observed in the panniculus carnosus and adventitia, even at low concentrations. The venom may penetrate the layers and be transported into the circulation by the lymphatic system [31]. This would explain the development of myotoxicity after the progression of necrosis, even given the short fang length [5]. As in the study of Iddon et al., even when the venom was injected intradermally, it penetrated deep into the layers of the skin, which could result in skeletal muscle injury [7].

Some animal studies have investigated the administration of antivenom to prevent venom-related dermonecrosis [5,7]. In Taiwan, the antivenom administered to patients who have been bitten by *N. atra* is a bivalent freeze-dried neurotoxic antivenom (FNAV) against *B. multicinctus* and *N. atra* [2]. The equine antivenom induced by the injection of crude venom from *N. atra* may not induce the production of adequate antibodies against the CTXs. In this study, we determined the MND of the deNTXs, and the MND_50_ could not be determined, which meant that the antivenom did not effectively neutralize the CTXs. In the study by Wu et al., the neutralization efficacy of antivenom was poor for CTXs A2, 4 and 5 [18]. As mentioned above, CTXs play a major role in necrosis. The polyvalent antivenom generated in horses after a challenge with crude venom cannot neutralize the CTXs, which might explain why it is ineffective at preventing necrosis. Other antivenoms also have little to no effect with regard to local damage [9,32,33]. To prevent necrosis after a patient has been bitten by a snake in the *Naja* genus, monoclonal antibodies against for CTXs [18,34,35] or combined treatment with other therapies [36] could be considered in the future.

The study of Dr. Liu et al. also discussed the venom effect of *Naja atra* [9]. We both used the deNTX model to present the human wound in the animal models and concluded that there was no effective neutralization of the antivenom for the necrotic progression. In our study, except the deNTX, we analyze the role of CTXs and PLA_2_ in the necrosis condition. We clarified that CTXs played the major role in the necrotic changes. We also highlighted that the venom went deeper to cause necrosis even though the dose was less than MND and the noneffective of antivenom anti-necrosis based on the tissue biopsy scored by an animal pathologist.

### Limitations

There are several limitations of this study. Although we removed the toxins one-by-one in this study, we could not rule out the possibility of a synergistic effect of NTXs on necrosis. The antivenom concentration could not be increased in this animal experiment; however, in clinical practice, patients may respond to increased doses of antivenom [6].

## 4. Conclusions

The use of the deNTXs allows mice to survive long enough to develop venom-induced cytolytic effects. CTXs play a major role in *Naja atra* venom-induced necrosis. The MND_50_ could not be identified in this study, which meant that the antivenom did not prevent necrosis. In the future, management of *Naja atra* bites may include not only the existing antivenom to improve the survival rate but also the administration of monoclonal antibodies against the CTXs or combined treatment with other therapies.

## 5. Materials and Methods

### 5.1. Chemical Reagents and Samples

Trifluoroacetic acid (TFA), ammonium bicarbonate (ABC), formic acid (FA), dithiothreitol (DTT), iodoacetamide (IAM), and Tween 20 were purchased from Sigma-Aldrich (St. Louis, MO, USA). Sodium hydroxide, DMSO, iodomethane and trichloromethane were purchased from Merck Millipore (Darmstadt, Germany). Trypsin was purchased from Promega (15,664 unit/mg, Sequence Grade, WI, USA). Acrylamide, SDS and TEMED were obtained from Bio-Rad (Hercules, CA, USA). Acetonitrile (ACN) was purchased from J.T. Baker (Phillipsburg, NJ, USA). Deionized water was generated with a Simplicity Ultrapure Water System (Millipore, Burlington, MA, USA) with a measured value of 18 MΩ.

### 5.2. Snake Venom Approach and Analysis

Venom was collected from 10 healthy adult specimens of *N. atra*. Each specimen was manually restrained, and the venom was milked. The liquid venom samples were individually obtained, lyophilized and stored at −80 °C until use. Commercial bivalent equine antivenom intended for clinical usage and antivenin against the venom of *B. multicinctus* and *N. naja atra* (Trade name: Antivenin of *B.* *multicinctus* and *N.* *naja* *atra* Antivenin Bivalent (lyophilized), 1000 antivenom unit/vial; batch number: 61-06-0010) were produced from the Taiwan Centers for Disease Control (CDC).

### 5.3. Preparation of deNTXs, deNTXs-deCTXs, and deNTXs-dePLA_2_

The lyophilized crude *N. atra* venom was dissolved in water and centrifuged at 10,000× *g* for 10 min. The amount of protein in the venom was determined via a BCA Protein Assay kit (Pierce^TM^, Thermo Scientific). The supernatants were diluted and further purified by size exclusion chromatography. The purified venom proteins, NTXs, were isolated from the crude venom following the procedure described by Huang et al. [18]. All the venom protein components other than the NTXs were combined and dissolved in PBS, creating the deNTXs. The crude venom and deNTXs were loaded onto a Phenomenex Jupiter^®^ C18 (250 × 4.6 mm, 5 µm particle size, 300 Å pore size) column with an ultraperformance liquid chromatography (UPLC) system (LC-20ADXR, Shimadzu, Kyoto, Japan) equipped with a DAD detector (SPD-M20A, Shimadzu, Kyoto, Japan) and autosampler (SIL-20ACXR, Shimadzu, Kyoto, Japan). The venom components were eluted at 1 mL/min with a linear gradient of 0.1% TFA in water (Solvent A) and 0.1% TFA in 100% ACN (Solvent B) (2% B for 5 min, followed by 2–10% B for 2 min, 10–16% B for 6 min, 16–28% B for 2 min and 28–65% B for 37 min) [18]. Protein elution was monitored at 215 nm (absorption wavelength for peptide bonds). The relative abundance (expressed as the percentage of the total venom protein) of each protein family was estimated as described by Huang et al. [18].

### 5.4. Minimum Necrotizing Dose (MND)

According to the WHO Expert Committee on Biological Standardization [24], the MND of venom is the smallest amount of venom (in μg of dry weight) that, when injected intradermally into the dorsal skin of lightly anesthetized mice, leads to the development of necrotic lesions 5 mm in diameter 3 days later. CD1 mice (10–12 weeks old) were obtained from BioLASCO Taiwan Co. Ltd. (Taipei, Taiwan) and grouped randomly (6 mice/group). The animal handling protocol was reviewed and approved by the Institutional Animal Care and Use Committee (IACUC) of the National Defense Medical Center (IACUC-20-112). One group of mice underwent removal of the fur from the dorsal skin and the intradermal injection of 50 µL of sterile saline; these mice served as the control group. Next, the mice in the testing groups underwent removal of the fur from the dorsal skin and a single intradermal injection of deNTXs (16.5, 20.5, 25.5, 32, and 40 µg). The diameter of the necrotic area of dorsal skin was measured after 72 h. The MND was determined with linear interpolation.

### 5.5. MND_50_: Neutralization Efficacy of Antivenom

The MND_50_ is a measure of the ability of an antivenom to prevent venom-induced dermonecrosis [24]. The MND_50_ is identified as the dose of antivenom (in microliters) that results in a diameter of the necrotic lesion that is 50% smaller than that of the lesion induced by the injection of venom and saline [24]. Two times the MND of deNTXs was selected as the challenge dose. The antivenom was bivalent antivenom against *B. multicinctus* and *N. atra,* which was manufactured by the National Health Research Institutes, Miaoli, and distributed by the Taiwanese Center for Disease Control, Taiwan, R.O.C. [1]. A fixed dose of venom was incubated with various dilutions of antivenom for 30 min at 37 °C. The positive control was venom incubated with saline instead of antivenom. Then, aliquots of 0.5 mL of the mixtures containing an amount of venom corresponding to two times the MND were injected intradermally into groups of six CD1 mice (10–12 weeks old).

### 5.6. Biopsy and Necrosis Score

The mice were intradermally injected with deNTXs (0.5, 0.33, 0.22, 0.148, and 0.098 µg/g) or 2 times the MND with different dilutions of antivenom (from the original: 1:2, 1:3, 1:4 and 1:5) into the dorsal skin. After 72 h, the mice were euthanized, and the dorsal skin was removed and sent for skin biopsy with hematoxylin and eosin (HE) staining. Then, the biopsy was scored by an animal pathologist based on the necrosis (Table 2).

### 5.7. Statistical Analysis

We report the data as the means ± standard deviations (SDs). Statistical analysis was performed by t-test, one-way ANOVA and Bonferroni’s multiple comparisons test using Prism 6 (GraphPad Software, Inc., San Diego, CA, USA). Statistical differences were considered significant when *p* was ≤ 0.05.

## Figures and Tables

**Table 1:** Minimum necrotizing dose (MND) in different components of *Naja atra*.

Name	Retained Toxin	Concentration (mg/mL)	Toxin Weight (µg)	MND (µg/g)
deNTXs	PLA_2_, CTX, others ^1^	0.198 ± 0.012	9.88 ± 0.576	0.494 ±0.029
deNTXs-dePLA_2_	CTX, others ^1^	0.118 ± 0.020	5.89 ± 1.005	0.294 ± 0.050
deNTXs-deCTXs	PLA_2_, others ^1^	>>0.5	>>25	>>1.25

Abbreviations: NTX, neurotoxin; PLA_2_, phospholipase A_2_; CTX, cytotoxin also called cardiotoxin. ^1^: CRISP, cysteine-rich secretory protein; HMWP, high-molecular-weight proteins.

**Table 2:** Tissue necrosis score.

Necrosis Score	Severity	Description
0	Normal	Within normal limits
1	Minimal	Sporadic occurrence
2	Mild	Aggregated distribution
3	Moderate	Regional distribution
4	Severe	Diffuse distribution and lose originality

## Data Availability

The data presented in this study are available from the corresponding author upon reasonable request.

## Supplementary Materials

The following are available online at https://www.mdpi.com/article/10.3390/toxins13090619/s1, Supplementary Data include: Table S1: Necrosis scores with different deNTXs doses (each group contained six mice). Table S2: Necrosis scores under a fixed challenge dose (2MND) mixed with different dilution times of antivenom (AV) (each group contains five mice).
