# Supplementary material for: Analysis of the Necrosis-Inducing Components of the Venom of Naja atra and Assessment of the Neutralization Ability of Freeze-Dried Antivenom
Source: Toxins (Basel). 2021 Sep 2;13(9):619. doi: 10.3390/toxins13090619 (PMC8473173; doi:10.3390/toxins13090619)
Supplement: Supplementary file 1 [file toxins-13-00619-s001.zip › toxins-1328604-supplementary.pdf]

toxins

Supplementary Materials: Analysis of the Necrosis-Inducing Components of the Venom of *Naja atra* and Assessment of the Neutralization Ability of Freeze-Dried Antivenom

Cheng-Hsuan Ho, Liao-Chun Chiang, Yan-Chiao Mao, Kuo-Cheng Lan, Shih-Hung Tsai, Yu-Jen Shih, Yuan-Sheng Tzeng, Chin-Sheng Lin, Wen-Loung Lin, Wei-Hsuan Fang, Kuang-Ting Chen, Chi-Hsin Lee, Dapi-Meng Lin Chiang, Shing-Hwa Liu *

Table S1. Necrosis score with different D-NTX doses (each group contain six mice).

deNTXs dosage	0.5 μg/g						0.33 μg/g						0.22 μg/g						0.148 μg/g						0.098 μg/g					
Epidermis	3	0	4	0	4	3	4	4	3	3	0	0	3	4	4	0	0	4	3	3	0	0	2	0	0	0	0	3	0	0
Dermis	3	0	4	0	4	4	4	4	4	4	0	0	3	4	4	0	2	4	3	3	0	0	3	0	0	0	0	3	0	0
Hypodermis	3	0	4	0	4	4	4	3	3	3	0	0	4	4	4	0	3	4	4	3	0	0	3	0	0	0	0	3	2	1
Panniculus carnosus	4	4	4	4	4	4	4	4	4	4	3	3	4	4	3	4	4	4	4	3	3	3	4	4	3	3	1	4	2	2
Adventitia	3	3	4	4	4	4	4	4	4	4	4	4	4	4	4	3	4	4	4	3	2	2	4	4	2	2	1	3	3	3

Table S2. Necrosis score under fixed D-NTX doses (2MND) and different dilution times of AV (each group contain five mice).

Antivenom	A1					A2					A3					A4					A5					A6				
Epidermis	3	0	3	3	3	3	0	4	4	0	0	3	0	0	0	4	3	3	1	0	0	4	4	2	0	0	3	3	0	3
Dermis	3	0	4	3	4	3	0	4	4	0	0	3	0	0	0	4	3	3	1	0	0	4	4	0	0	0	3	3	0	3
Hypodermis	3	2	3	3	4	3	0	4	4	0	0	3	0	0	0	2	3	2	2	0	0	4	4	3	0	0	3	3	0	3
Panniculus carnosus	4	4	4	4	3	3	0	3	3	3	0	0	4	4	4	2	4	4	3	3	4	3	3	4	4	2	3	3	4	3
Adventitia	2	2	4	3	3	2	0	4	4	3	0	1	4	4	3	0	0	1	3	3	3	4	4	3	3	2	3	3	3	3

A1: original AV concentration; A2: 1:1 dilution ; A3: 1:2 dilution ；A4: 1:3 dilution ；A5: 1:4 dilution ；A6: 1:5 dilution.
